# Harnessing the Therapeutic Potential of the Nrf2/Bach1 Signaling Pathway in Parkinson’s Disease

**DOI:** 10.3390/antiox11091780

**Published:** 2022-09-09

**Authors:** Manuj Ahuja, Navneet Ammal Kaidery, Debashis Dutta, Otis C. Attucks, Eliot H. Kazakov, Irina Gazaryan, Mitsuyo Matsumoto, Kazuhiko Igarashi, Sudarshana M. Sharma, Bobby Thomas

**Affiliations:** 1 Darby Children’s Research Institute, Medical University of South Carolina, Charleston, SC 29406, USA; 2 Department of Pediatrics, Medical University of South Carolina, Charleston, SC 29406, USA; 3 vTv Therapeutics LLC, High Point, NC 27263, USA; 4 Fordham University, Bronx, NY 10452, USA; 5 Pace University, White Plains, NY 10601, USA; 6 Department of Chemical Enzymology, School of Chemistry, M.V. Lomonosov Moscow State University, 111401 Moscow, Russia; 7 Faculty of Biology and Biotechnologies, Higher School of Economics, 111401 Moscow, Russia; 8 Department of Biochemistry, Graduate School of Medicine, Tohoku University, Sendai 980-8576, Japan; 9 Department of Biochemistry & Molecular Biology, Medical University of South Carolina, Charleston, SC 29406, USA; 10 Hollings Cancer Center, Medical University of South Carolina, Charleston, SC 29406, USA; 11 Department of Neuroscience, Medical University of South Carolina, Charleston, SC 29406, USA; 12 Department of Drug Discovery, Medical University of South Carolina, Charleston, SC 29406, USA

**Keywords:** Parkinson’s disease, Nrf2, Bach1, antioxidants, oxidative stress, electrophile

## Abstract

Parkinson’s disease (PD) is the second most common neurodegenerative movement disorder characterized by a progressive loss of dopaminergic neurons in the substantia nigra pars compacta. Although a complex interplay of multiple environmental and genetic factors has been implicated, the etiology of neuronal death in PD remains unresolved. Various mechanisms of neuronal degeneration in PD have been proposed, including oxidative stress, mitochondrial dysfunction, neuroinflammation, α-synuclein proteostasis, disruption of calcium homeostasis, and other cell death pathways. While many drugs individually targeting these pathways have shown promise in preclinical PD models, this promise has not yet translated into neuroprotective therapies in human PD. This has consequently spurred efforts to identify alternative targets with multipronged therapeutic approaches. A promising therapeutic target that could modulate multiple etiological pathways involves drug-induced activation of a coordinated genetic program regulated by the transcription factor, nuclear factor E2-related factor 2 (Nrf2). Nrf2 regulates the transcription of over 250 genes, creating a multifaceted network that integrates cellular activities by expressing cytoprotective genes, promoting the resolution of inflammation, restoring redox and protein homeostasis, stimulating energy metabolism, and facilitating repair. However, FDA-approved electrophilic Nrf2 activators cause irreversible alkylation of cysteine residues in various cellular proteins resulting in side effects. We propose that the transcriptional repressor of BTB and CNC homology 1 (Bach1), which antagonizes Nrf2, could serve as a promising complementary target for the activation of both Nrf2-dependent and Nrf2-independent neuroprotective pathways. This review presents the current knowledge on the Nrf2/Bach1 signaling pathway, its role in various cellular processes, and the benefits of simultaneously inhibiting Bach1 and stabilizing Nrf2 using non-electrophilic small molecules as a novel therapeutic approach for PD.

## 1. Parkinson’s Disease, a Heterogeneous Neurodegenerative Movement Disorder

Parkinson’s disease (PD) is the most common movement disorder that affects nearly 10 million people worldwide. The PD-patient population mainly comprises a segment of the elderly population above sixty. Furthermore, PD is the second most common neurodegenerative disease globally, ranking behind Alzheimer’s disease in prevalence and adverse social/economic impact. The United States alone is estimated to have approximately 1.2 million PD patients by 2030, which will cost 52 billion U.S. dollars annually in direct and indirect costs related to treatment, social security payments, and lost income [1,2]. In 1817, James Parkinson provided the first description of clinical cases of PD in his seminal essay, “Essay on the shaking palsy”, and even more than two centuries later, PD remains a condition with uncertain etiopathogenesis. Clinically, PD consists of four cardinal motor symptoms—commonly known as parkinsonian symptoms—that include rigidity, resting tremor, bradykinesia (slowness in movement), and postural instability resulting in gait dysfunction (reviewed in [3]).

Additionally, non-motor manifestations of PD include gastrointestinal dysfunction, cognitive impairment, depression, anxiety, hyposomnia, sleep disorders, and autonomic dysfunction. Interestingly, these non-motor symptoms appear long before the onset of motor-related symptoms [4,5]. Although most PD cases’ etiological factors are unknown and are described as sporadic PD, approximately 10–15% of all PD cases consist of a monogenic form of PD with a well-defined single causative genetic mutation. Currently, at least 23 loci and 19 disease-causing genes for PD have been found [6]. Although sporadic PD has no definitive etiological factors, recent population-based studies have identified distinct genetic risk factors that interact with well-known environmental factors in PD [3].

Pathologically, PD involves the loss of nigrostriatal dopaminergic neurons associated with the cytoplasmic accumulation of α-synuclein inclusions in neuronal cell bodies (Lewy bodies, LBs) and neuronal processes (Lewy neurites, LNs). Other cell types throughout the central and peripheral autonomic nervous system are also known to accumulate α-synuclein inclusions. Various neurodegenerative pathways and mechanisms have been proposed based on the experimental evidence that has elucidated genetic and environmental risk factors for PD. The neurodegenerative pathways implicated in PD are proteinopathy (accumulation of α-synuclein in Lewy bodies, dysfunction of the proteasome, and autophagy), oxidative stress (OS), mitochondrial dysfunction, neuroinflammation, disruption of calcium homeostasis, and other cell death pathways. While many drugs targeting these pathways have shown promise in preclinical PD models, this promise has not yet translated into neuroprotective therapies in human PD. Therefore, considerable effort has been focused on identifying novel targets for facilitating drug discovery in PD treatment. In the present review, we harness the therapeutic potential of nuclear factor E2-related factor 2 (Nrf2), a key transcription factor orchestrating the cell response to OS, and its target gene BTB and CNC homology 1 (Bach1), a transcriptional repressor of Nrf2, which hold great promise in modulating multiple etiological pathways against neuronal cell death in PD.

## 2. Role of Oxidative Stress in PD Pathogenesis

OS is defined as the excessive production of reactive oxygen species (ROS) and reactive nitrogen species (RNS) in conjunction with the reduced antioxidant capacity that leads to changes in the cellular redox balance. Physiologically, the cellular redox balance (equilibrium), also known as redox homeostasis, is achieved via enzymatic and non-enzymatic elimination of highly reactive oxidative species continuously generated over a lifetime [7]. Redox homeostasis plays a cardinal role in maintaining physiological signaling pathways involved in host defense, gene transcription, regulation of neuronal plasticity [8], apoptosis, and cellular homeostasis [7]. Electrophilic oxygen- and nitrogen-centered free radicals and anions (ROS and RNS) constitute cellular OS as the unavoidable byproducts of mitochondrial respiration and some anabolic and catabolic reactions. The cellular antioxidant arsenal includes both antioxidant enzymes (e.g., superoxide dismutase [SOD], catalase [CAT], glutathione peroxidase, glutathione-S-transferase, etc.) and stoichiometric radical quenchers such as melatonin, glutathione, and carotenoids. The antioxidant enzymes provide highly reducing intracellular conditions [9] with the ratio of reduced glutathione (GSH) to oxidized glutathione (GSSG) as an indicator of cellular health, with the reduced GSH constituting up to 98% of cellular GSH under normal conditions [10] and thus keeping the steady-state concentration of intracellular superoxide and hydrogen peroxide below nanomolar concentrations [11]. However, under pathological conditions, the OS overwhelms the existing antioxidant system’s capacity and damages macromolecules such as lipids, proteins, and DNA, ultimately resulting in neuronal death [7,12]. The central nervous system (CNS) is especially vulnerable to OS due to several factors. First, the CNS’s high metabolic demand generates a ten-fold higher amount of ROS than any other tissue. Second, neurons contain insufficient catalase and rely mainly on the thiol-reducing system for quenching hydrogen peroxide. Third, the CNS has many lipids, which readily react with ROS to generate toxic oxidative agents such as aldehydes. ROS but not reactive nitrogen species (RNS)-induced modifications of proteins can be repaired by the thioredoxin reductase system and peroxiredoxins. However, the lipid-derived aldehyde coupling to proteins is an irreversible reaction and non-repairable with reducing enzymes [13,14]. One of the reactive dopamine oxidation intermediates, 3,4-dihydroxyphenylacetaldehyde (DOPAL), can covalently modify proteins leading to protein aggregation, OS, and cell death. Moreover, α-synuclein proteostasis, a recurrent element in PD pathology, is considered a preferential target for DOPAL [15]. Detoxification of aldehydes depends on the activity of aldehyde dehydrogenase (ALDH) [16], and specific activators of this enzyme are under development as a therapeutic strategy, but this topic is beyond the scope of this review. However, mitochondrial ALDH2 is a known Nrf2 target [17], as well as ALDH1A1 and ALDH3A1 family members [18], and thus, general activation of the Nrf2 pathway will detoxify harmful aldehydes produced under OS conditions.

### 2.1. Sporadic PD

**Figure 1:**
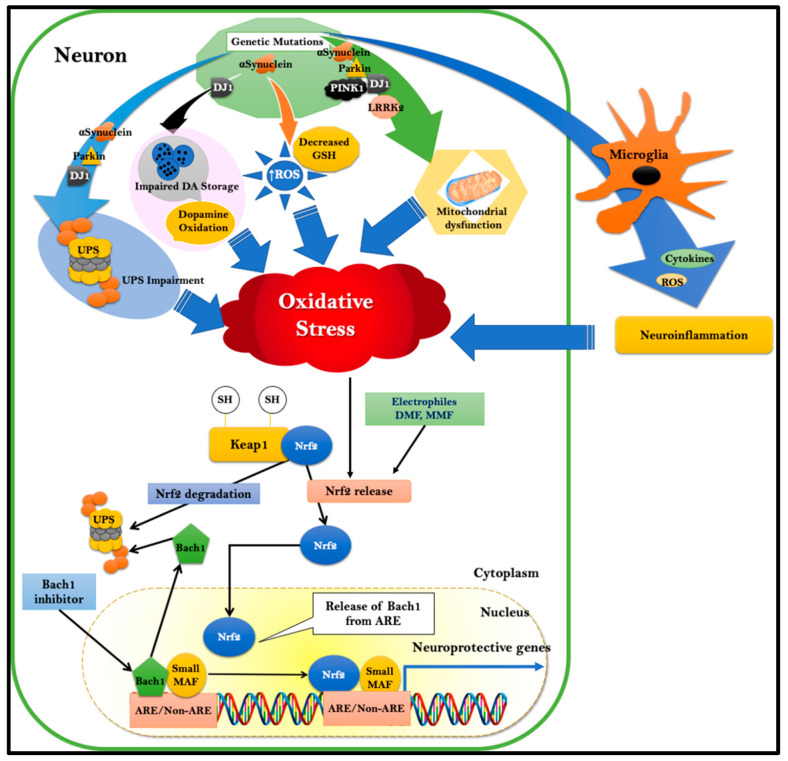
Cartoon showing the dysregulation of oxidative stress-mediated pathways in PD and the utility of the Nrf2/Bach1 signaling pathway as a therapeutic target. Mitochondrial dysfunction, proteasomal impairment, oxidative stress, and neuroinflammation are the key players underlying Parkinson’s pathophysiology. Both environmental factors and genetic mutations of DJ-1, Parkin, PINK1, α-synuclein, and LRRK2 increase oxidative stress, which results in neuronal cell death. Oxidative stress activates the Nrf2 pathway by covalently modifying the Keap1-Nrf2 interaction and releasing Nrf2 to translocate into the nucleus freely. Once in the nucleus, Nrf2 forms heterodimers with the small MAF protein, and this competitively displaces Bach1 from the ARE binding site. This results in the transcriptional activation of antioxidant, anti-inflammatory, and neuroprotective genes that work in unison against PD-associated neurodegenerative pathways. Electrophilic Nrf2 activators can also disrupt the Keap1-Nrf2 interaction and result in the activation of the Nrf2 pathway. Unfortunately, these Nrf2 activators also deplete the GSH levels and contribute to oxidative stress in the neurons, which are already in a compromised state. Bach1, a physiological repressor of Nrf2, occupies the same binding site as Nrf2. Bach1-inhibition by genetic and pharmacological means results in the activation of the Nrf2 pathway, subsequently imparting neuroprotection in PD pathogenesis. The Bach1 inhibitor (HPPE) is a well-established non-electrophile and does not deplete GSH and thus does not contribute to oxidative stress.

OS has been proposed to play a significant role in aging and neurodegenerative disorders such as PD [19,20]. Although causal relationships between oxidative damage and PD remain to be defined fully, the evidence has shown that oxidative damage occurs in PD as indicated by oxidative markers of lipid, protein, and DNA in postmortem tissues. Substantia nigra (SN) neurons from postmortem PD brains show increased levels of 8-hydroxyguanine (8OHG), a marker of oxidative DNA damage [21], and an increase in 4-Hydroxy-2-nonenal (HNE) immunoreactivity, a marker of lipid peroxidation, when compared to controls [22]. Nigrostriatal dopaminergic neurons are particularly vulnerable to OS compared to non-dopaminergic neurons as they are intrinsically “equipped” with ROS-generating components such as the neurotransmitter dopamine (DA) [15] and neuromelanin and have high iron content. Under basal conditions, primary antioxidants such as GSH are crucial in scavenging ROS and minimizing oxidative burdens. However, the levels of GSH in SN are significantly reduced before the initiation of dopaminergic neurodegeneration, as seen in incidental Lewy body disease [23]. In addition, the downregulation of the primary enzymatic antioxidant protein, SOD in SN, is also confirmed in the data collected from preclinical models and human postmortem PD brains [24,25]. The loss of initial antioxidant defense mechanisms is accompanied by dysfunctional mitochondrial electron transport chain activities, specifically complex I. This results in the loss of mitochondrial membrane potential and the amplified generation of OS [26,27]. These sequential events significantly hamper mitochondrial ATP production in cells. In addition, a lack of ATP compromises DA uptake into synaptic vesicles, resulting in an abundance of DA in the cytosol. DA, when present at neutral pH in the cytosol, has a high propensity for undergoing spontaneous oxidation in the presence of oxygen and iron. A significant increase in cysteinyl adducts of L-DOPA, DA, and DOPAC has been found in the substantia nigra of PD patients, suggesting the cytotoxic nature of DA oxidation products [28]. DA quinones can cyclize to form a highly reactive and stable o-quinone aminochrome that polymerizes with neuromelanin and mediates the generation of superoxide and the depletion of the cellular NADPH [20,29]. Neuromelanin and other oxidized and nitrated molecules released from degenerating neurons likely contribute to a self-destructive degenerative process in healthy DA neurons by affecting the functions of neighboring glial cells such as astrocytes and microglia (Figure 1).

In a healthy brain, astrocytes maintain ion homeostasis, provide structural and metabolic support, and regulate synaptic transmission and blood flow. On the other hand, microglia continuously extend and retract their process to interact with neurons and other types of glial cells, including astrocytes [30]. Therefore, astrocytes are proposed to remove aggregated proteins and work in coordination with microglia to keep the brain microenvironment clean [31]. Neuron-released α-synuclein oligomers are shown to activate glial cells via the interaction with Toll-like receptor-2 (TLR-2), resulting in the activation of the NF-κB-mediated inflammatory cascade and generation of inflammatory molecules such as IL-1β, TNF-α, IL-6, etc. [32]. At the same time, the generation of anti-inflammatory tissue-repairing cytokines, such as IL-10 and TGF-β, is generated in lesser amounts by the activated pro-inflammatory microglia [33,34]. The shift towards pro-inflammatory M1 microglia in the PD brain also correlates with high levels of superoxide-producing NADPH oxidases (NOX). The primary function of NOX is to generate ROS, which is believed to be important in the CNS host defense and in the redox signaling circuits that shape the different activation phenotypes of the microglia [35]. The role of microglia in OS was supported by the findings from the Przedborski laboratory, which reported that postmortem SN samples from sporadic PD patients had higher NOX2 protein content compared to healthy controls. They showed that NOX2 immunostaining is localized with the hyperactive microglia but not with the substantia nigra pars compacta (SNpc) DA neurons [36]. Consistent with microglial NOX2 upregulation and postmortem detection of elevated levels of ROS in the brain of PD patients, microglia-induced ROS is strongly implicated in DA neurodegeneration by compromising the homeostatic support of glial cells to DA neurons. On the other hand, microglia-derived inflammatory mediators such as IL-1α, TNF-α, and C1q induce A1 astrocytes that have more deleterious effects on neurons [37,38]. In addition, toxic protein oligomers are known to directly travel from donor astrocytes to recipient healthy astrocytes via the tunneling nanotubes to cause mitochondrial damage in the recipient cells [39]. The existence of glia-derived inflammatory substances is confirmed by their presence in the brains and cerebrospinal fluid of patients with PD [37,40].

Various molecular mechanisms have been proposed in the selective degeneration of DA neurons of the SNpc in PD. One possible contributing factor is their massive, unmyelinated axonal arbor combined with the high bioenergetic cost for their maintenance, which is orders of magnitude greater than other brain neuronal subtypes [41]. The dopaminergic neurons of SN are metabolically more active. They have a higher rate of mitochondrial oxidative phosphorylation and an elevated level of basal OS compared to the neighboring ventral tegmental area (VTA) neurons [42]. This observation is confirmed by higher transcript levels of mitochondrial proteins such as ND1, prohibitin, and triosephosphate isomerase in SNpc DA neurons [43,44]. The enhanced dependency on the mitochondrial bioenergetic pathways for survival makes the nigral dopaminergic neurons more vulnerable to environmental toxins and mutant gene products than the VTA subtypes. Apart from being the energy source, mitochondria are dynamic organelles regulating calcium homeostasis, stress response, and cell death. In this context, the mishandling of calcium by DA neurons plays a significant role in OS. DA neurons in SNpc are autonomously active through their pace-making activity [45]. This pace-making activity is driven by voltage-dependent L-type calcium channels, leading to sustained elevations in cytosolic calcium levels in the SNpc DA neurons. The enormous calcium-buffering burden created by pace-making compromises mitochondrial function, resulting in mitochondrial OS. Interestingly, the VTA DA neurons, which do not rely on L-type calcium channels for pace-making, are relatively spared in PD [46]. Corroborating evidence for the selective vulnerability of SNpc DA neurons to OS comes from studies that measured the intracellular redox state of DA neurons in brain slices using a redox-sensitive green fluorescent protein to demonstrate that in normal conditions without ongoing pathology, DA neurons in the SNpc are more oxidized than those from the VTA [47]. Furthermore, differential labeling of oxidized and reduced cysteines to determine the global thiol/disulfide redox state in DA neurons at the single cell level confirmed a higher oxidation status in SNpc DA neurons compared to neurons from the VTA [48]. These observations suggest that an imbalance in redox homeostasis in SNpc DA neurons, coupled with mitochondrial dysfunction and the interference of pro-inflammatory mediators secreted from activated glial cells, generates OS, which predisposes neurons to PD-linked pathology.

### 2.2. Familial PD

The discovery of genes linked to familial forms of PD has yielded important insights into the molecular pathways in disease pathogenesis and highlighted mechanisms by which OS contributes to the disease (Figure 1).

#### 2.2.1. α-Synuclein

Mutations in the gene encoding α-synuclein (PARK 1 and 4) have been associated with familial variants of PD with autosomal dominant inheritance [49]. Missense point mutations on the N-terminal (A53E, A53T, A30P, E46K, H50Q, and G51D) region of the α-synuclein gene cause autosomal dominant PD. In contrast, the duplication and triplication of the α-synuclein gene cause early-onset familial PD [50]. Pathogenic mutations and elevated concentrations give α-synuclein a propensity to develop a β-sheet structure that readily polymerizes into oligomers and higher-order aggregates such as fibrils, which are a common feature of PD and α-synucleinopathies. The aggregation of α-synuclein is observed not only in the midbrain dopaminergic neurons but also in the gut, olfactory bulb, and hippocampus of individuals even before the onset of SNpc DA neurodegeneration in the PD [51,52,53].

Several studies suggest a direct pathologic relationship between OS and α-synucleinopathies. Specifically, Lewy bodies in human α-synucleinopathies are associated with an accumulation of the oxidatively modified form of α-synuclein, including nitrated α-synuclein [54]. Oxidative damage to α-synuclein, particularly dityrosine cross-linking of α-synuclein, is associated with increased aggregation [55,56,57]. The transition of α-synuclein from protofibrils to mature fibrils is inhibited by the oxidative modification of α-synuclein by DA, resulting in the accumulation of cytotoxic protofibrils in DA neurons, suggesting that catechol oxidation may contribute to the accumulation of α-synuclein [58]. It is suggested that pore-forming α-synuclein protofibrils can disrupt the integrity of vesicular membranes, thereby increasing cytosolic catecholamine concentrations further and exacerbating the toxicity of oxidized catechols [59,60]. Accordingly, human-induced pluripotent stem cell-derived DA neurons from a PD patient carrying an α-synuclein triplication show high levels of OS markers with increased susceptibility to hydrogen peroxide, suggesting that the α-synuclein overexpression intrinsically changes the balance of ROS production and DA neuron survival [61]. Several reports demonstrate α-synuclein’s direct role in OS-induced neurodegeneration is due to its role in mitochondrial dysfunction. The translocation of α-synuclein in the mitochondria was shown in experimental PD models by overexpression of the wild-type or mutated form of the protein [62,63]. Out of three major mutations, the A53T and E46K forms of α-synuclein have shown maximum penetration through the mitochondrial membranes [64,65,66,67]. In contrast, the A30P mutation reduces the translocation of α-synuclein into the mitochondria [65,68]. This observation is explained by the increased intra-protein hydrogen bonds and stability in the case of A53T. It is also defined by eliminating the negative charge in the case of E46K in the N-terminal region, which might augment the entry of α-synuclein through the lipids in the mitochondrial outer membrane. Apart from that, several pieces of evidence, including protein–protein interaction studies, also support the binding of α-synuclein with some essential mitochondrial membrane proteins, including the voltage-dependent anion channel (VDAC) [69], the translocase of the outer membrane 40 (TOM40) [70], or the protein transporter TOM20 [71]. On a functional basis, in vivo and in vitro studies in midbrain dopaminergic neurons suggested that α-synuclein predominantly accumulates in the inner mitochondrial membrane and interacts with the complex I, resulting in the impaired activity of the mitochondrial electron transport chain [72,73,74,75]. α-synuclein-induced neurodegeneration can also be mediated through microglia activation leading to an increased ROS generation [73]. A proposed mechanism of excessive microglial activation and a subsequent pro-inflammatory state in the brain is suggested by the release of aggregated α-synuclein from dying DA neurons. Notably, oligomeric α-synuclein-induced microglial activation leads to elevated ROS production followed by the secretion of pro-inflammatory cytokines [76,77,78]. These findings suggest that both genetic and biochemical abnormalities in α-synuclein result in OS, whereas OS is sufficient to cause α-synuclein aggregation and DA neuron degeneration.

#### 2.2.2. LRRK2

Mutations in the leucine-rich repeat kinase 2 (LRRK2, PARK8) gene are associated with the autosomal dominant late-onset PD [79]. LRRK2 belongs to the ROCO protein family and contains tandem Ras-of-complex proteins (Roc), GTPase, and C-terminal of Roc (COR) domains characteristic of this family [80]. Mutations in this gene manifest in 5–6% of familial cases and approximately 1.6% of sporadic PD cases worldwide [81,82]. Three major mutations in this gene are reported in different populations, including G2019S and I2020T on the kinase domain and R1441C occurring in the protein’s ROC/COR GTPase domain [83,84]. Of the LRRK2 mutations, the G2019S mutation in its kinase domain is the most frequently observed mutation that leads to increased kinase activity. A greater vulnerability of LRRK2 mutant cells to OS is linked to the kinase-dependent interaction between LRRK2 and dynamin-like protein (DLP1), which facilitates DLP1 translocation to the mitochondria and subsequent mitochondrial fission [85,86]. Overexpression of wild-type or mutant LRRK2 with enhanced kinase activity in various cell lines or primary neurons leads to mitochondrial fragmentation, mitochondrial DNA (mtDNA) damage, and dysfunction associated with increased ROS generation and increased susceptibility to hydrogen peroxide [85,87,88]. Mutations in the LRRK2 kinase domain increase the phosphorylation of mitochondrial thioredoxin-dependent peroxide reductase, peroxiredoxin 3 (PRDX3), causing the inhibition of the enzyme’s activity and exacerbating OS-induced neuronal death. Notably, postmortem brains from PD patients carrying the G2019S mutation show a marked increase in phosphorylated PRDX3 compared to controls [89]. Additionally, markers of OS (8-hydroxy-2′-deoxyguanosine and 8-isoprostane) are increased with a corresponding decrease in total antioxidant content in the cerebrospinal fluid of healthy individuals with PD-associated LRRK2-G2019S mutation [90]. This observation is consistent with findings from Drosophila and transgenic mice, where the G2019S mutation has been shown to impart an increased sensitivity to mitochondrial toxins and OS [91,92], and a similar response has been reported in induced pluripotent stem cell-derived DA neurons from LRRK2 mutation carriers [93]. Overall, these observations indicate that LRRK2-activating mutations will compromise the detoxification of peroxides and increase OS, thus leading to mitochondrial dysfunction. Another possibility by which mutated LRRK2 imparts a toxic effect on mitochondria and other organelles is by promoting the synthesis and aggregation of mutant α-synuclein and a decreased removal of oligomers through autophagy. Direct evidence comes from the studies where mutant LRRK2 was shown to potentiate the accumulation and toxicity of α-synuclein in A53T transgenic mice. In contrast, A53T α-synuclein toxicity was blocked in the LRRK2 knockout mice [94]. The hypothesis behind this may lie either in the enhancement of α-synuclein translation from the mRNA promoted by the LRRK2 [95] and the enhanced extracellular signal-regulated kinase pathway [96] or in the reduced removal of α-synuclein oligomers caused by the decreased chaperone-mediated autophagy in the presence of mutated LRRK2 [97]. Notably, activation of the Nrf2-signaling mitigates toxicity induced by α-synuclein and LRRK2 by promoting protein homeostasis in a cell-autonomous and time-dependent fashion. Nrf2 accelerates the clearance of α-synuclein, shortening its half-life, leading to lower levels of α-synuclein. By contrast, Nrf2 promotes the aggregation of LRRK2 into inclusion bodies, leading to a significant reduction in diffuse and mutant LRRK2 levels into more insoluble and homogenous inclusion bodies [98].

#### 2.2.3. Parkin

Loss of function mutations in the Parkin gene (PARK2) cause an autosomal recessive early-onset PD [99]. Parkin is an E3 ligase and functions as a multipurpose neuroprotective protein against various toxic insults [100]. Multiple reports suggest that parkin’s neuroprotective action is mediated via the modulation of OS. Neuronal cells overexpressing wild-type parkin have low levels of ROS and are protected against apoptosis caused by DA [101]. Contrary to this, the expression of mutant parkin is associated with increased OS markers such as protein carbonyls, lipid peroxidation, and nitrated proteins [102]. Induced pluripotent stem cells derived from DA neurons in patients carrying parkin mutations show increased OS markers and enhanced activity of Nrf2 [103,104]. Notably, the activity of parkin is known to be impaired by oxidative and nitrosative stress-induced post-translational modifications consistent with the presence of DA-quinone-modified parkin in the SN of PD patients [105,106,107]. Parkin exerts its protective action against OS by removing damaged mitochondria that cause increased ROS generation. Under physiological conditions, parkin is localized to the cytosol. However, upon OS, parkin translocates to the mitochondria and removes the depolarized and damaged mitochondria by mitophagy [108]. Further studies confirmed that the activation of parkin is PINK-1 dependent, and the phosphorylation of parkin mediates this activation at Ser65 in both ubiquitin and the N-terminal ubiquitin-like domain [109]. Once activated, parkin triggers a cascade of events, including Drp1-mediated fission of damaged mitochondria, ubiquitination of proteins present in the mitochondria, and proteins such as Mfn1 and Mfn2 to hinder the fusion process and the targeting of damaged mitochondria for mitophagy. Proper regulation of mitophagy is crucial for maintaining cellular homeostasis, and inadequate removal of damaged mitochondria through mitophagy leads to the generation of ROS [110]. However, there are pathways beyond mitochondrial dynamics that are regulated by parkin. Both nuclear respiratory factors 1 and 2 and peroxisome proliferator-activated receptor γ coactivator 1α (PGC-1α) play indispensable roles in mitochondrial biogenesis since they activate the gene expression of several respiratory chain complex proteins. Parkin is reported to promote the ubiquitin-dependent degradation of the parkin-interacting substrate (PARIS/ZNF746), a KRAB and zinc finger protein, and a transcriptional repressor of PGC-1α [111,112]. In PD models, PARIS accumulates due to a loss of parkin, leading to downregulation of PGC-1α and impairment of the mitochondrial biogenesis [111]. Dysregulation of PGC-1α alters redox homeostasis and mitochondrial biogenesis, whereas increasing PGC-1α levels protect against OS, restores mitochondrial biogenesis, and prevents the loss of nigral DA neurons [111,113,114,115]. These reports suggest a reciprocal relationship between parkin function and OS and regulation of mitochondrial quality control in PD pathogenesis where OS activates parkin to remove damaged mitochondria, and loss of parkin function causes mitochondrial dysfunction and increases OS.

#### 2.2.4. PINK1

Mutations in the PINK1 (PARK6) gene, a mitochondrial-targeted serine-threonine kinase, cause autosomal recessive early-onset PD [116]. PINK1 is a crucial regulator of mitochondrial quality control, preserving mitochondrial respiration involved in mitochondrial transport and cell death, possibly via the phosphorylation of its substrates. Mitochondrial protein phosphorylation is involved in cell stress-induced apoptosis and contributes to mitochondrial dynamics and mitophagy regulation. The link between OS and PINK1 is potentially attributed to the actions of PINK1 on mitochondria, which serve as a significant source of ROS. PINK1 deficiency results in the shortening, swelling, and fragmentation of mitochondria in cultured cells [117,118,119,120] and is associated with the loss of mitochondrial complex I activity [120]. Flies lacking PINK1 function show fragmented mitochondria with imprecise cristae morphology accompanied by reduced mitochondrial membrane potential, reduced respiration, and reduced ATP production in their spermatids, indirect flight muscles, and loss of DA neurons [121,122,123]. PINK1 null mice exhibit an increase in the number of giant mitochondria and impaired mitochondrial respiration, showing increased sensitivity to OS [124]. Consistent with these findings, induced pluripotent stem cells (iPSC) from PINK1 mutant human subjects show increased vulnerability to the complex I inhibitor and hydrogen peroxide. In contrast, the treatment of antioxidants can mitigate these abnormalities associated with PINK1 deficiency [125]. Under OS conditions, PINK1 expression is regulated by Nrf2 [126], and PINK1 is required to recruit Parkin to mitochondria and initiate mitophagy to scavenge the damaged mitochondria [127,128]. Consistent with this observation, Nrf2 activation is sufficient to suppress OS, restore mitochondrial function, and elevate rates of mitophagy in Parkin or PINK1 deficient flies [129]. Besides the collaborative role of PINK1 with parkin in mediating mitochondrial dynamics, several studies report PINK1 to play a role independent of parkin in mitochondrial homeostasis. Investigations of PINK1 knockout Drosophila and mouse models demonstrate a specific role of PINK1 in regulating the activity of the mitochondrial complex I. PINK1-deficiency in these models leads to primary mitochondrial complex I defect [130] associated with the loss of phosphorylation of the complex I subunit, NDUFA10, at Ser250 [131]. An over-expression of NDUFA10 could restore the mitochondrial activity in PINK1-deficient conditions but not during parkin deficiency, suggesting that the effect of PINK1 on mitochondrial complex I via NDUFA10 is independent of parkin. These studies indicate that PINK1 is critical for maintaining mitochondrial homeostasis and protecting DA neurons against OS and mitochondrial dysfunction.

#### 2.2.5. DJ-1

Mutations in the DJ-1 gene are linked to the autosomal recessive early-onset PD [132]. The DJ-1 protein has multiple functions and exhibits the properties of a molecular chaperone, protease, glyoxalase, and transcriptional regulator that protects mitochondria from OS [133]. Different oxidation states of DJ-1 function as a cellular redox sensor, determining the cell fate by either activating autophagy or apoptosis by regulating the activity of ASK1, a member of the mitogen-activated protein kinase family that activates c-Jun N-terminal kinase and p38 in response to OS, endoplasmic reticulum stress, and calcium influx. The overexpression of DJ-1 promotes GSH synthesis and makes the cells resistant to OS-induced cell death, while lower levels of DJ-1 make the cells susceptible to OS (reviewed by [134]). In a study by Lopert and Patel, mitochondria isolated from the brains of DJ-1 knockout mice exhibited greater thioredoxin activity and GSH levels than the control. However, the decreased activity of glutathione reductase suggests an adaptive response of the mitochondria in the absence of DJ-1 [135]. More recent studies [136,137] link DJ-1 to the inhibition of ferroptosis, a unique form of programmed cell death characterized by the cytosolic accumulation of iron and currently considered a significant driver of cell death in neurodegenerative diseases [138,139]. DJ-1 is mainly localized in the cytoplasm, and under OS, it translocates to the mitochondria [140,141]. A conserved cysteine residue in DJ-1 (Cys106) can undergo oxidative modification, enabling DJ-1 to neutralize ROS [142]. Mild oxidation of Cys106 to the sulfinyl form (SO2H) is necessary for mitochondrial localization and the protection of cells against OS [143]. The N-terminal sequence of DJ-1 is essential for its mitochondrial localization [144], where it binds directly with the NDUFA4 and ND1 subunits of mitochondrial complex I [145]. DJ-1 gene deletion in human DA neurons leads to the depolarization and fragmentation of mitochondria along with the accumulation of autophagy markers in the vicinity of fragmented mitochondria. These damaging effects on mitochondria due to DJ-1 deficiency are abrogated by antioxidants, suggesting that the mitochondrial dysfunction in DJ-1 knockout cells was partly due to OS [146]. Mitochondrial ROS production can be reduced by mild uncoupling under low doses of protonophores that partially reduce the mitochondrial membrane potential [147]. Uncoupling of mitochondria by uncoupling proteins in the inner mitochondrial membrane prevents excessive ROS production. DJ-1 increases the expression of uncoupling protein (UCP)-4 via the NF-κB pathway, as DJ-1 enhances NF-κB translocation to the nucleus where NF-κB regulates transcriptional activity of UCP-4. On the other hand, the loss of DJ-1 results in the downregulation of the UCP-4 [148].

DJ-1 increases the expression of vesicular monoamine transporter 2 (VMAT2) [149,150] and keeps the cytoplasmic DA levels in check by enhancing the reuptake of DA into synaptic vesicles and protecting against DA toxicity and accumulation of ROS. DJ-1 also plays a crucial role in sensing and conferring protection against OS by exhibiting molecular chaperone, protease, and transcriptional regulator properties and protecting mitochondria from OS [133,134]. DJ-1 stabilizes Nrf2 by preventing its proteasomal degradation by Kelch-like epichlorohydrin-associated protein 1 (Keap1) and induces the expression of antioxidant genes, leading to the reduction of ROS [151]. The main conclusions from the study of familial PD gene products support the existence of crosstalk between mitochondrial dysfunction and OS that can lead to DA neuron loss. They also point to the potential of the genetic program orchestrated by Nrf2 to serve as a promising therapeutic target against familial PD.

## 3. The Nrf2 Pathway as a Therapeutic Target for PD

Aging is a primary risk factor for PD, causing a significant decline in cellular antioxidant capability [152]. Hence, reinforcing cellular pathways to combat OS may represent a promising strategy for delaying the onset of PD and slowing neurodegeneration. One way to bolster cellular antioxidant pathways is to activate Nrf2 signaling, which acts as the primary genetic program for cellular defense against OS, inflammatory reactions, and toxic electrophiles. Under basal conditions, Nrf2 is sequestered by Keap1 in the cytoplasm, facilitating the ubiquitination and proteasomal degradation of the Nrf2 [153,154]. However, under OS conditions or in the presence of an electrophile, cysteine moieties in Keap1 are oxidized, allowing Nrf2 to circumvent proteasomal degradation and enabling its nuclear translocation. In the nucleus, Nrf2 acts as a trans-activator to express genes that harbor cis- antioxidant response element (ARE) in their promoter sequence [155]. Nrf2 signaling regulates nearly 250 genes involved in the antioxidant, anti-inflammatory responses, mitochondrial bioenergetics, [156], and other cellular repair pathways [157]. In addition to its well-known role as a master regulator of the antioxidant defense, Nrf2 serves as a hub that compiles signals derived from misfolded protein accumulation to build a coordinated transcriptional response to maintain the endoplasmic reticulum integrity and protein homeostasis [158].

Several lines of evidence suggest the impairment of the Nrf2 pathway in PD [156,159]. For instance, preclinical and clinical evidence illustrates a significant decline in Nrf2 activity in aging [160,161], which is a considerable risk factor for the development of PD. Moreover, post-mortem PD brains exhibit increased nuclear localization of Nrf2 with an increased expression of ARE-driven genes, including NADPH quinone oxidoreductase 1 (Nqo1) and heme oxygenase (HO-1) in the surviving dopaminergic neurons. This suggests an insufficient neuroprotective counterattack to oxidative damage [162]. Similarly, identifying a functional haplotype in the Nrf2 promoter that renders constitutive activation of the Nrf2 pathway has been shown to confer a decreased risk and a delayed onset of PD in two groups of European PD patients [163]. These findings suggest that Nrf2 dysregulation is implicated in the pathogenesis of PD and that activation of Nrf2 may be an effective therapeutic target.

Multiple drugs are currently being tested in clinical trials for PD [164]. Among these, only Tocovid Surabio (tocotrienol-rich Vitamin E from palm oil) is in phase II clinical trial and can be classified as an antioxidant with some capacity to active Nrf2 [164]. The SURE-PD3, a Phase III clinical trial, evaluated the potential of inosine, a precursor of uric acid (UA), to slow PD progression [165]. UA is an endogenous purine metabolite and accounts for most of the antioxidant capacity in the blood. Increasing epidemiological and clinical evidence has supported the view that higher UA levels are associated with a decreased risk and a slower disease progression in PD [166,167]. The presumptive neuroprotective action of UA includes scavenging oxygen radicals, chelating iron, blocking iron-dependent oxidation reactions, slowing the DA auto-oxidation rates, stabilizing calcium homeostasis, preserving mitochondrial function, and activating the Nrf2 pathway [168,169]. Various cellular and animal preclinical PD models have shown therapeutic effects of both UA and inosine (reviewed in [170]) by activating the Nrf2 pathway. However, despite the beneficial effects of inosine and UA in preclinical PD models, a good safety profile in humans, and epidemiological evidence in slowing disease progression in PD, the SURE-PD3 trial was unsuccessful [165]. In our hands, neither inosine nor UA activated the Neh2-luciferase reporter (Figure S1), a direct screen for Nrf2 protein stabilizers working via disruption of the Nrf2-Keap1 axis [171]. Hence, the failure of the SURE-PD3 trial cannot be considered a failure of the Nrf2 activation strategy in PD since both inosine and UA are not direct Nrf2 activators.

Indeed, comprehensive endeavors employing Nrf2 activators in preclinical PD models have yielded substantial neuroprotection [172,173,174,175]. Nevertheless, these Nrf2-activators are highly electrophilic and do not appear to be suitable candidates for PD therapy [176]. Our work with synthetic triterpenoids (TP-319 and TP-500), highly potent Nrf2 activators, has shown that these agents are neuroprotective in the 1-methyl-4-phenyl-1,2,3,6-tetrahydropyridine (MPTP) mouse model of PD [177]. Unfortunately, these synthetic triterpenoids are highly cytotoxic at higher doses [177], demanding extreme caution in their therapeutic application. We and others have analyzed the FDA-approved small-molecule Nrf2 activator, dimethyl fumarate (DMF), in both in vitro and in vivo models of PD. These reports suggest that DMF is a potent antioxidant, anti-inflammatory, and neuroprotective agent and improves mitochondrial functions [173,178,179,180]. However, DMF acutely depletes GSH levels and causes neuronal death at higher doses in vitro [173]. Additionally, multiple reports corroborate our findings regarding the side effect profile of DMF. It includes flushing, gastrointestinal disturbances, and progressive multifocal leukoencephalopathy, limiting DMF’s utility as a PD therapeutic [181]. As we demonstrated, DMF’s bioactive metabolite, monomethylfumarate (MMF), exhibits a similar therapeutic profile to DMF with fewer side effects [173]. Therefore, based on our observations, we hypothesize a strategic advantage for targeting the Nrf2 signaling in PD, although this approach still warrants extreme caution in clinical settings. Nonetheless, utilizing pharmacological Nrf2 activators remains contentious as a clinical trial of bardoxolone methyl, a potent triterpenoid-based Nrf2 activator, was terminated because of adverse effects, including death in a patient [182,183].

To overcome the limitations of electrophilic Nrf2-activators, novel therapeutic strategies must be identified and developed. One approach is to formulate non-electrophilic peptides or small molecule-based protein–protein interaction inhibitors (PPIs) that disrupt Keap1-Nrf2 interactions by binding to specific Keap1 motifs that interact with Nrf2 [176,184]. Thus, these PPIs act as competitive non-covalent activators of Nrf2. However, to date, the PPIs that have been developed show mild potencies in vitro and in vivo when compared to the electrophilic Nrf2-activators [185]. Furthermore, besides releasing Nrf2 from Keap1, PPIs may also displace other Keap1 client proteins from the ubiquitin ligase complex causing off-target effects unless designed to ensure specificity for the Kelch domain of Keap1 [185]. Hence, further work is necessary to develop more potent and selective PPIs without off-target effects. A second novel strategy is to search for an alternative target to activate the Nrf2 pathway without involving the electrophilic modification of cellular proteins. The transcriptional repressor Bach1 is one such target that serves as a physiological repressor of Nrf2 and modulates both Nrf2-dependent and Nrf2-independent protective responses.

## 4. Bach1: Structural Domains, Expression, and Transcription-Dependent and Independent Roles

Bach1 belongs to one of the 19 phylogenetically related families of the basic region leucine zipper containing transcription factors. It is evolutionarily and closely linked to the cap “n” collar (CNC)-related basic-region leucine zipper transcription factor family that includes p45 NF-E2, Nrf1/LCR-F1/TCF11, Nrf2/ECH, and Nrf3 [186]. The Bach family is a relative newcomer in the evolutionary tree of bZIP proteins. It is found only in higher eukaryotes except for *Ciona intestinalis*, implicating that Bach proteins play a role in maintaining the complex functions of life in the higher eukaryotes [186]. At the same time, Bach1 is unique from Nrf2 and other CNC-related proteins because it contains a combination of two vastly distinctive domains: (1) Broad complex, tram track, bric-a-brac/poxvirus, and zinc finger (BTB/POZ) and (2) CNC-related bZip leucine fingers [187,188]. The BTB/POZ domain acts as a protein-interaction motif facilitating self-association and heterodimeric interaction with non-BTB proteins [189]. BTB family proteins, which include BTB-zinc finger (ZF), Skp1, and Elongin C, regulate diverse physiological roles that include transcriptional repression [190], cytoskeletal regulation [191,192], tetramerization, and the gating of ion channels [193], and the facilitation of ubiquitination [194].

**Figure 2:**
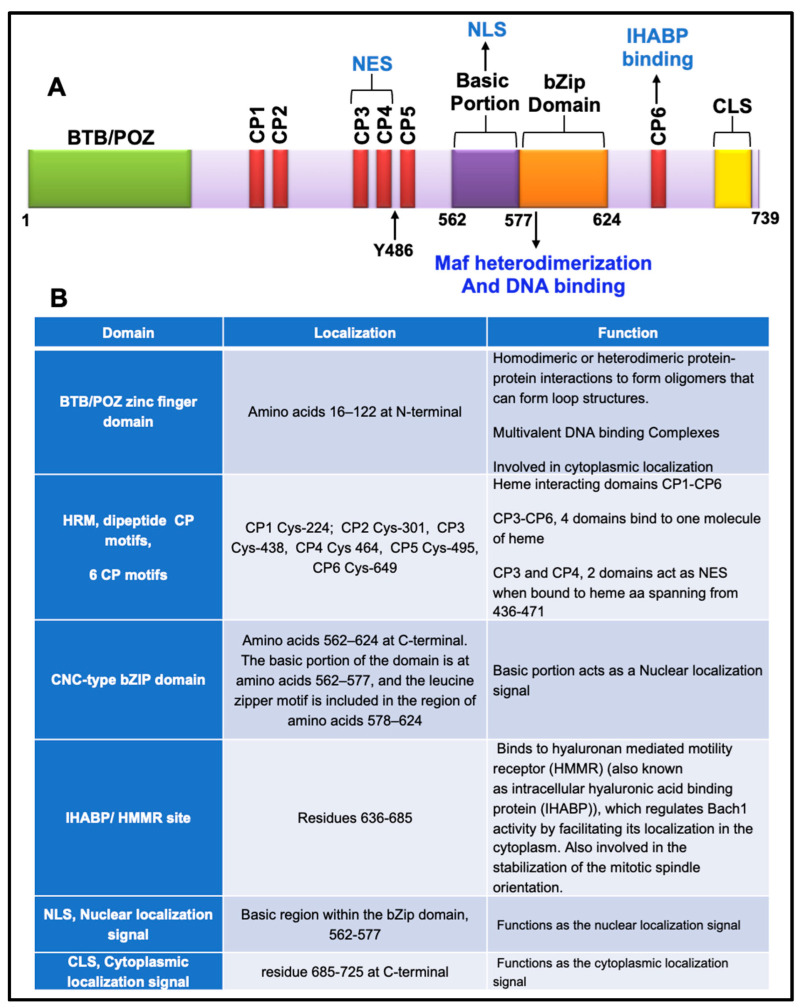
(**A**) Pictorial representation of mouse Bach1 protein structure with different domains. (**B**) Table illustrating the different functional domains present in the Bach1 protein.

As mentioned above, Bach1 possesses BTB/POZ and CNC-related bZip leucine finger domains [188]. Furthermore, Bach1 has multiple other domains with distinct functional roles [188,195,196,197,198]. Based on the functional role, the structure of Bach1 is categorized into five domains: (1) The BTB/POZ zinc finger domain (amino acids 16–122) at the N-terminal, (2) the heme regulatory motif (HRM) composed of dipeptide cysteine-proline (CP 1–6) recurring at six positions, (3) a CNC-type bZIP domain extending from amino acids 562–624, (4) an intracellular hyaluronic acid binding protein (IHABP/HMMR) site residing in the region encompassing residues 636–685, and (5) a cytoplasmic localization signal (CLS) composed of residues spanning 685–725 (Figure 2).

### 4.1. Bach1 Expression and Transcriptional Regulation

Human Bach1 is ubiquitously expressed in all mammalian tissues [188]. In mice, Bach1 is expressed at high levels in hematopoietic cells, bone marrow cells, the thymus, and the liver from embryonic day 13.5 onwards. It is also expressed in the brains of adult mice. One of the earliest events in hematopoietic cell development is the induction of Bach1 [199]. The human Bach1 gene is localized on chromosome 21q22.1 (HC21) [200] and consists of five exons. The Bach1 promoter consists of three regions: (1) An upstream negative regulatory region, (2) a minimal core promoter region, and (3) a stimulatory region that resides between the first two regions. The minimal core promoter region exhibits the two well-conserved GC boxes that bind to a commonly expressed *trans*-acting DNA binding factor, SP1 (specificity protein 1). The basal expression of Bach1 is under the control of SP1 [201]. The same study showed that Bach1 alone indirectly represses its transcription through an unknown mechanism. However, it is shown to be mediated by the ARE site in the Bach1 promoter region [201]. The Bach1 promoter has two ARE sequences, ARE1 and ARE2, at +1411 and +1270 downstream of the TSS (transcription start site). The Nrf2 activating compounds induce Bach1 expression via the ARE1 site located at +1411 in an Nrf2-dependent fashion [202,203]. In addition to transcriptional regulation, based on the different domains of Bach1 protein (Figure 2), it is evident that its subcellular localization and cellular function are affected by various stimuli.

### 4.2. Transcription-Dependent and -Independent Mechanisms of Bach1

**Figure 3:**
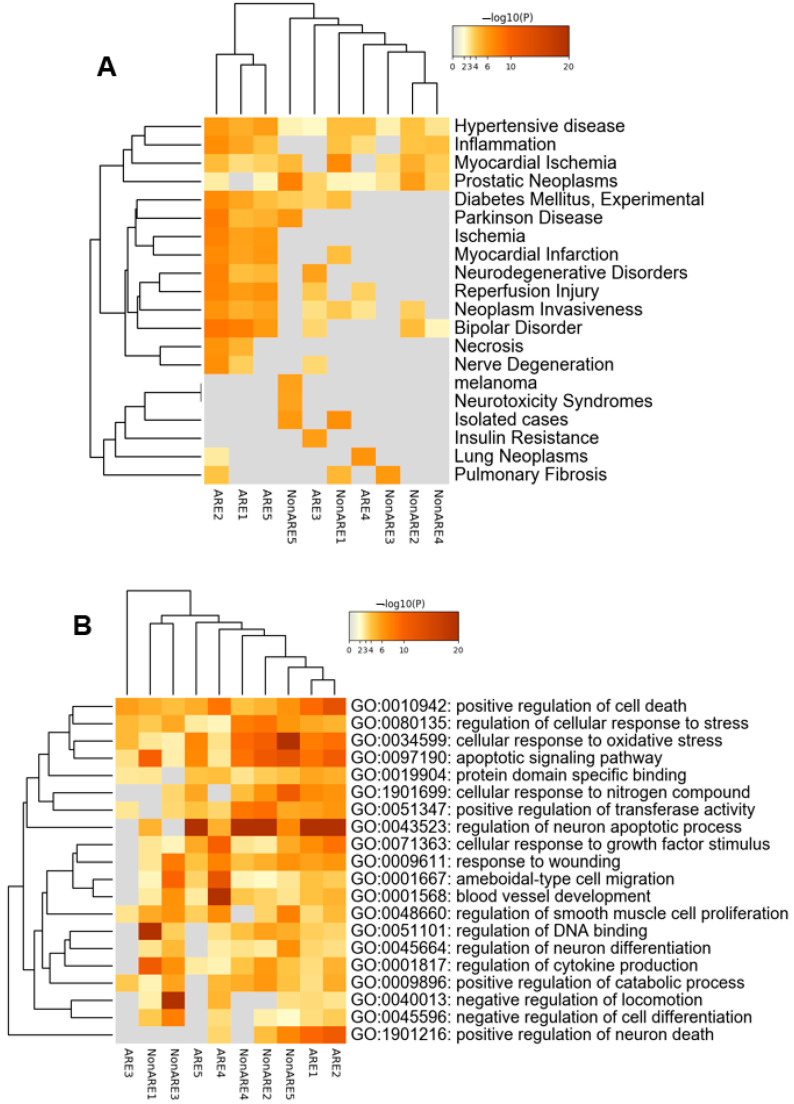
(**A**) Enrichment of disease terms by gene signatures that contain either ARE or non-ARE genes. (**B**) Heatmap of enriched biological processes in ARE and non-ARE containing gene signatures.

As a transcription factor, Bach1, similar to other CNC-related proteins, heterodimerizes with small musculoaponeurotic fibrosarcoma (MAF) proteins to form a dimeric or oligomeric complex that binds to small Maf responsive element (MARE) consensus sites on the DNA. Once bound to the corresponding DNA-binding elements, small MAF-Bach1 heterodimers act as a transcriptional repressor by recruiting methionine adenosyltransferase II (MATII), which methylates the DNA and histone and epigenetically represses gene expression [204,205]. Recent studies also implicate the interaction of Bach1 with proteins other than small MAF proteins in occupying DNA motifs dissimilar to MARE-like sequences and exhibiting MAFK-independent DNA binding [206]. Therefore, it is highly probable that the Bach1 deletion may activate Nrf2/MAFK-independent targeted genes. Indeed, our functional genomics analysis has revealed 1154 differentially expressed genes in the VMB region between the wild-type and Bach1 knockout mice. By using the motif analysis of previously published Bach1 ChIP-seq data and our microarray data, we have identified both ARE and non-ARE gene expression profiles [207]. To our surprise, 52% of 2242 Bach1-associated genes have non-ARE motifs. Pathway enrichment analyses (Metascape) [208] demonstrate that ARE-associated genes are enriched for pathways involved in oxygen sensing/regulation and neuronal death (Figure 3). In contrast, non-ARE-bound genes primarily affect DNA binding, inflammatory response, apoptosis, and neuronal death (Figure 3). Recently, the CRISPR/Cas9 mediated Bach1 knockout in a pancreatic cancer cell line (AsPC1) shows novel Bach1 targeted genes such as CLDN3, CLDN4, PKP2, CHD1, and FOXA1, a subset of genes that enhance the metastasis of tumor cells [209]. Interestingly, most Bach1 target genes contain ARE-like motifs in the Bach1 binding regions. However, gene expression of others involved additional mechanisms besides Bach1. For example, the authors observed that CDH1 expression was indirectly regulated by Bach1. It is thought that Bach1 inhibits the expression of CDH1 by repressing FOXOA1 and activating SNAI2, the repressor and activator genes of CDH1, respectively. However, the double knockdown of Bach1 and FOXOA1 did not change CDH1 expression. These findings suggest that the transcriptional regulation of Bach1 is not only a direct effect but also a secondary mechanism involving additional transcription factors that likely play a role in gene expression.

Recent advances in elucidating Bach1 function have demonstrated that it also exhibits transcription-independent roles. A series of reports from Li et al. [192,210,211] has shed light on the transcription-independent role of Bach1 in facilitating chromosomal alignment during mitosis. Mitosis-specific phosphorylation of Bach1 at various sites switches its primary function from transcription to mitosis, which, along with HMMR and CRM1, stabilizes the orientation of the mitotic spindle [211]. Together, these findings suggest that Bach1 is a relatively new protein that holds enormous promise for future investigations into its role in normal physiology and pathophysiological conditions.

## 5. Role of Bach1 as a Mediator of Cellular Redox Homeostasis

Recent evidence has suggested that Bach1 plays a vital role in maintaining cellular redox homeostasis. Investigative studies involving the knockdown and knockout of Bach1 have demonstrated that these manipulations significantly increase the expression of HO-1, an Nrf2 target gene. It has been shown that Nrf2 cannot bind to the ARE site at the promoter region of the HO-1 gene in the presence of Bach1, suggesting the crucial role of Bach1 in repressing the HO-1 expression [212]. HO-1 is a rate-limiting enzyme involved in heme catabolism. Degradation of heme yields iron, carbon monoxide, and biliverdin, which is transformed into bilirubin under the catalytic effect of biliverdin reductase. Heme degradation products are all biologically active molecules mainly implicated in tissue redox homeostasis to mitigate OS and counteract its adverse effects [213,214]. HO-1 is pivotal in regulating redox homeostasis because of its anti-inflammatory, antioxidant, and anti-apoptotic properties. Besides its role as a rate-limiting enzyme in heme catabolism, HO-1 has several non-canonical functions, including chaperone activity mediated by protein–protein interactions, transcriptional regulation, intracellular compartmentalization, mitochondrial bioenergetics, and immunomodulation [215,216]. Furthermore, considerable evidence proves that HO-1 induction renders therapeutic effects in animal models of various disorders [reviewed in [214]].

Bach1 possesses four critical cysteine-proline (CP) motifs at its C-terminal that are instrumental in heme binding [217,218]. Therefore, one significant role of Bach1 is to maintain free heme levels in the cytoplasm by regulating the expression of various genes involved in hemoglobin synthesis [219], iron metabolism [204,205], and heme elimination [218]. On the same grounds, due to several cysteine residues in its integral protein structure, Bach1 responds to alterations in the cellular redox states. Accordingly, it acts as a cellular redox regulator [220]. Additionally, similar to other cellular redox sensors, well-known sulfhydryl oxidizing agents such as diamide [220] and other electrophiles, including cobalt protoporphyrin [221], cadmium [195], and HNE, modulate Bach1 activity [220]. Furthermore, Kitamuro et al. demonstrate the hypoxia-induced repression of HO-1 induction in three human cell lines by modulating Bach1 expression [222]. Hence, this evidence strongly implicates Bach1 in modulating cellular redox potential and maintaining cellular homeostasis.

Indeed, the approach of modulating Bach1 activity to attenuate OS response has been successfully used in various preclinical models of OS-mediated disorders. Notably, Bach1-deficient mice show an anti-atherosclerotic effect in high-fat diet-fed apolipoprotein E (Apo E) knockout mice, which are highly susceptible to the deposition of atherosclerotic plaques in the blood vessels. The suppression of atherosclerotic plaque in Bach1-deficient mice is abolished by tin protoporphyrin (SnPP), an HO-1 inhibitor, implicating HO-1 dependency in the protective phenotype [223]. The same group also demonstrates the suppression of neointimal formation after cuff injury in Bach1-deficient mice, which was mediated by the reduced proliferation of vascular smooth muscle cells (SMC) and increased phagocytic activity compared to wild-type mice [224]. Furthermore, Mito et al. show the cardioprotective effect of Bach1 ablation in an in vivo model where transverse aortic constriction results in left ventricular (LV) hypertrophy and remodeling, ultimately leading to heart failure. Bach1 gene deletion prevented LV hypertrophy, fibrosis, and wall thickening and maintained the left ventricle’s contractile function [225].

Similarly, the Bach1 gene deletion has shown antioxidant, anti-apoptotic, and anti-inflammatory effects against the indomethacin-induced intestinal injury model [226,227] and the lipopolysaccharide (LPS) and D-galactosamine (GalN) induced hepatotoxicity [228]. Bach1 knockout mice are protected against 2,4,6-trinitrobenzene sulfonic acid (TNBS)-induced colitis in which isolated peritoneal macrophages have characteristics of the M2 state (a state involved in immunosuppression and tissue repair) and exhibited a high level of HO-1 expression [229]. An investigation into the effect of Bach1 ablation on osteoarthritis reveals significant protection against age-associated and surgically induced osteoarthritis [230]. Interestingly, the same study shows that the increased HO-1 expression because of Bach1-ablation and the subsequent antioxidant effects are crucial in imparting a protective effect against osteoarthritis [230]. Furthermore, RANKL is shown to induce the nuclear translocation of Bach1, which neutralizes the Nrf2-driven cellular antioxidant capability and mediates the osteoclastogenic effect of RANKL. Thus, in an in vivo model of bone destruction, nuclear export of Bach1 by increasing heme levels following administration of 5-aminolevulinic acid (ALA) and ferrous citrate exhibits a protective effect [231]. The same group showed that pharmacological Bach1 inhibition effectuated RANKL-mediated intracellular ROS and subsequently attenuated the RANKL-mediated bone resorption [232]. Thus, Bach1 ablation has beneficial effects against cardiovascular dysfunction, hepatotoxicity, intestinal toxicity, and osteoarthritis via the modulation of redox homeostasis.

## 6. Role of Bach1 as an Immunomodulator

Innate and acquired immunity plays a crucial role in maintaining an organism’s normal state of homeostasis. The resulting inflammatory response is a natural process in repairing tissues and the organism’s defense against infections and harmful agents. However, the chronic ectopic stimulation of immune pathways can lead to auto-inflammatory damage to the cellular system, eventually resulting in several disorders. The transcription factor Nrf2 has been demonstrated to inhibit the maturation of dendritic cells (DCs) in vitro and modulate immune responses [233]. Indeed, a well-known Nrf2 activator, dimethyl fumarate (DMF), exerts neuroprotective and anti-inflammatory effects against multiple sclerosis (MS), an autoimmune-related disorder [234,235], and is currently being clinically employed in treating MS [235]. Being a member of the same family of transcription factors as Nrf2 and as Nrf2’s competitive repressor of ARE sites, Bach1 is expected to play an essential role in innate and acquired immunity. Recent studies have established the crucial role of Bach1 in hematopoiesis [236,237,238], and it is thus speculated to be a critical regulator of the immune system. A recent investigation depicts the role of Bach1 in regulating steady-state myelopoiesis, normal immune function, and the development of autoimmune disorders [239]. The study results showed that Bach1 gene deletion does not cause any changes in the percentage of T cells, B cells, natural killer cells, and erythrocytes. However, the population of macrophages and DCs is significantly reduced compared to controls. Moreover, Bach1 ablation results in partial protection in the murine experimental autoimmune encephalomyelitis (EAE) model of MS, which was shown to be mediated via a defective T-cell response due to the impaired development of antigen-presenting cells (APCs) such as macrophages and DCs [239]. This effect of Bach1 gene deletion depends on HO-1 induction. More recently, conditional deletion of Bach1 in endothelial cells attenuated atherosclerosis by reducing endothelial inflammation [240]. In human and mouse atherosclerotic plaques, Bach1 was upregulated in the endothelial cells. Endothelial Bach1 gene deletion decreased atherosclerotic lesions, macrophage content in plaques, expression of endothelial adhesion molecules ICAM-1, VCAM-1, and reduced plasma TNF-α and IL-1β levels in atherosclerotic mice.

Bach1 and Bach2 are known to affect B-cell development, as Bach1 and Bach2 are a part of the gene-regulatory network that finetunes the balance between innate immunity and acquired immunity [238]. The most notable immune response-linked genes targeted by Bach1 include HO-1, IL-6, and MCP-1, all of which have well-known immunomodulatory effects [241,242]. A few studies have reported robust expression of Bach1 in neonatal lung tissue. Bach1 ablation is found to be protective against hyperoxic lung injury in newborn mice [242,243]. The underlying protective mechanism involved Bach1-inhibition mediated upregulation of HO-1 and IL-6 expression concomitant with the transient overexpression of proinflammatory cytokine MCP-1 [241]. Besides, a deficiency in Bach1 ameliorates 2,4,6-trinitrobenzene sulfonic acid (TNBS)-induced colitis by modulating the development of macrophages and APCs [229]. The study shows that Bach1 deficiency promotes the M2-type macrophages, which induce an extensive anti-inflammatory response upon injection into the TNBS-treated wild-type mice, as demonstrated by the reduced expression of pro-inflammatory markers such as TNF-α, IFN-γ, and KC in the colon [229]. Bach1 plays a regulatory role in the pathophysiology of lupus nephritis (LN), particularly in determining the pro-inflammatory or anti-inflammatory phenotype of M2 macrophages by modulating the HO-1 expression [244]. Recently, Pradhan et al. demonstrated that bone-marrow-derived macrophages show reduced mitochondrial oxidative phosphorylation and increased glycolysis resulting in the activation of the NLRP3 inflammasome [245]. Isolated bone-marrow-derived macrophages from Bach1 knockout mice showed enhanced mitochondrial membrane potential and generation of mitochondrial ROS along with reduced levels of mitophagy compared to wild-type, despite significant upregulation of HO-1 in the Bach1 knockout bone-marrow-derived macrophages. These findings suggest that Bach1 is a regulator of cellular bioenergetics with functional consequences for immunomodulatory activities characterized by the coexistence of both pro-inflammatory inflammasome activation and anti-inflammatory effects due to high levels of HO-1 due to Bach1 deficiency in macrophage activation. While mitochondrial dysfunction and NLRP3 activation were observed in isolated bone-marrow-derived macrophages from Bach1 knockout mice in culture, it is unknown whether such a mechanism exists in vivo in other cell types or tissues from the Bach1 knockout mice. Furthermore, the significance of these findings in modulating tissue inflammation, mitochondrial dysfunction, and cellular pathology in Bach1 knockout mice is currently unclear. A recent study by Cai et al. demonstrated that systemic and tissue inflammation was attenuated in Bach1 knockout mice along with improved organ function and survival following polymicrobial sepsis induced by cecal ligation and puncture (CLP). Besides attenuating tissue inflammation, the absence of Bach1 reduced mitochondrial dysfunction after CLP-sepsis by preserving bioenergetics function in mitochondria isolated from the liver of CLP-sepsis-induced mice. Gene expression profiling by RNA-seq analysis in the liver of Bach1 knockout mice compared to wild-type mice subjected to CLP-sepsis showed that the most significantly affected genes were predominantly associated with lipid metabolism, oxidoreductase activity, and significant upregulation in HO-1 expression. Although Bach1 ablation after CLP-sepsis preserved mitochondrial bioenergetics, the RNA-seq analysis failed to observe the upregulation of genes that encode proteins modulate mitochondrial bioenergetics in the Bach1 knockout mice. However, the inhibition of HO-1 activity by Zn protoporphyrin-9 worsened organ function in Bach1 knockout mice following CLP, suggesting a crucial role of HO-1 in these protective phenotypes in Bach1 knockout mice [246]. Many studies show the anti-inflammatory effects of Bach1 ablation in different OS-related disorders such as steatohepatitis and drug-induced hepatic and intestinal toxicity [226,247,248]. Hence, considering the existing knowledge stated above, it can be reasonably proposed that the Bach1/HO-1 axis plays a vital role in acquired and innate immunity. This also implies that the Bach1/HO-1 axis can be exploited as a drug target for auto-immune and metabolic disorders.

## 7. Bach1, a Modulator of Mitochondrial Bioenergetics and Iron Homeostasis

The Nrf2 pathway regulates the expression of various genes involved in maintaining the normal functionality of the mitochondrial bioenergetics [156]. Other investigators and we have elucidated the role of Nrf2 activation in regulating mitochondrial bioenergetics, metabolomics, and metabolic function, which we have previously discussed (reviewed in [156]). A subset of Bach1-targeted genes is mitochondrial-associated genes that modulate the diverse mitochondrial processes ranging from mitochondrial transcription and biogenesis to mitochondrial bioenergetics [249]. For instance, nuclear respiratory factor 1 (NRF1) is a transcriptional regulator of mitochondrial biogenesis, which has recently been shown to be targeted by Bach1 through secondary mechanisms, as NRF1 lacks Bach1 binding sites [249]. Additionally, an increasing number of studies implicate abnormally elevated Bach1 levels playing a central role in the cancer cell metastasis [209,250]. A previous study has demonstrated that Bach1 negatively regulates the expression of several mitochondrial electron transport chain-related genes with a concomitant increase in mitochondrial acidification and decreased mitochondrial respiration [251]. The same study identified six mitochondrial genes, ATP5D (also known as ATP5F1D), COX15, UQCRC1, ATP5J (also known as ATP5PF), SLC25A22, and TIMM8B, which Bach1 directly regulates as potential Bach1 target genes [251]. In another report, Weil et al. have identified genes associated with glycolytic pathways, such as Hexokinase-2 (HK-2) and Glyceraldehyde-3-Phosphate Dehydrogenase (GAPDH), which are under direct regulation by Bach1 [252]. These authors demonstrated that they could attenuate lung cancer metastasis by inhibiting the glycolytic pathways. These findings, combined with mitochondrial dysfunction in PD due to reduced mitochondrial respiration, dampened mitochondrial biogenesis, and elevated mitochondrial acidification, suggest a potential benefit of targeting Bach1 in attenuating neuronal damage associated with PD.

By repressing the HO-1 in various cell types, Bach1 modulates heme degradation and the recycling of iron, which is cytotoxic in its free form. A recent study on Bach1 ChIP-seq performed in human kidney cells (HEK293) has identified ferritin light chain (Ftl), ferritin heavy chain (Fth), and ferroportin as direct targets of the Bach1 repression [249]. These results are corroborated by those of Nishizawa et al., which demonstrated that Bach1 represses the transcription of a group of genes, including the glutamate-cysteine ligase modifier subunit (Gclm), solute carrier family seven-member 11 (Slc7a11), Fth1, Ftl1, and solute carrier family 40 member 1 (Slc40a1), all of which are involved in glutathione synthesis or cellular iron metabolism [205]. Additionally, Haldar et al. demonstrate that Bach1 represses Spi-C expression, a transcription factor known to regulate iron recycling in the macrophages [253]. Consequently, these findings indicate that Bach1 plays a vital role in maintaining iron homeostasis by controlling the various cellular steps involved in iron uptake, degradation, export, and reabsorption [205,254,255]. Thus, inhibiting Bach1 from modulating iron homeostasis may be beneficial in PD.

## 8. Does Bach1 Play a Role in PD?

As discussed above and reported in multiple studies within various pathologies, Bach1 plays a critical regulatory role in influencing the gene expression of several genes involved in redox regulation, mitochondrial biogenesis, and inflammation. Our functional genomic analysis reveals that Bach1 represses several Nrf2-mediated ARE and Nrf2-independent non-ARE genes and is involved in PD pathogenesis [207].

Our results also demonstrated that Bach1 is up-regulated in the SN of MPTP-treated mice and human postmortem PD brains. For the first time, these findings indicate that Bach1 up-regulation is associated with the demise of SNpc dopaminergic neurons of PD brains, as Bach1 knockout mice exhibited a significant reduction in MPTP-induced dopaminergic neuronal cell death in both acute and sub-acute models [207]. The attenuation of neuronal death in the SNpc is accompanied by the attenuated loss of dopamine and its metabolites in the striatum and reduced inflammation in the midbrain, as evidenced by the reduced expression of glial activation markers in Bach1 knockout mice compared to the MPTP-treated wild-type mice. Furthermore, gene expression analyses show that the ablation of Bach1 causes a significant up-regulation of 1164 genes in the VMB of mice. Bioinformatic analyses reveal that 33% of these differentially regulated genes harbor classic ARE motifs (TGA(G/C)TC) followed by the ETS motif (Table 1). Genes associated with Bach1-ARE motifs are enriched for pathways critical for oxygen sensing regulation and neuronal death. In contrast, genes enriched with non-ARE motifs were involved in DNA binding, inflammatory response, apoptosis, and neuronal death (Figure 3). Because the Maf family of transcription factors heterodimerizes with Bach1 and ETS transcription factors during differentiation and immune response [232,233,234,235], Bach1 could regulate non-ARE genes through ETS/MAF interactions. An important observation in our study is that a significant percentage of the up-regulated genes in the Bach1 knockout mice do not contain an ARE motif in their promoter regions and, therefore, belong to the non-ARE class of genes. This signifies that Bach1 might directly regulate the transcription of other genes, which are not involved in the OS response but possibly in different neuroprotective pathways. Most non-ARE genes regulated by Bach1 are represented by transcription factors and proteins that modulate neuronal cell survival. Therefore, it can be surmised that the ablation of Bach1 augments the endogenous expression of several cytoprotective genes. This finding is supported by studies demonstrating that Bach1 negatively regulates the expression of critical mitochondrial proteins such as ATP5D, NDUFA9, COX15, COX18, SLC25A15, UQRC1, and ATP5G3 [251] and preserves mitochondrial function during injury via HO-1 dependent mechanisms [246]. These proteins are essential for oxidative phosphorylation and the electron transport chain functions, cumulatively increasing glucose utilization through mitochondrial metabolism. Without Bach1, these proteins are upregulated to improve mitochondrial health. Our results suggest that Bach1 deficiency can upregulate both Nrf2 and non-Nrf2 genes, which may have additional benefits in PD. These results provide a strong rationale for further validating Bach1 as a therapeutic target in chronic and genetic PD models.

## 9. Small Molecule Therapeutics That Inhibit Bach1

The study by Ahuja and co-workers [207] convincingly demonstrates that Bach1 ablation has a pronounced neuroprotective effect in MPTP-induced parkinsonism and that Bach1 inhibition could serve as a drug target to prevent PD pathology. This necessitates the development of therapeutics to inhibit the physiological repressor function of Bach1 from activating the expression of cytoprotective genes. Hemin is a known physiological inhibitor of Bach1 DNA-binding activity and inducer of its nuclear export and degradation [196,198,218]. However, hemin also imparts a cytotoxic effect as, in its structure, the iron atom is coordinated by porphyrin ring pyrroles, which behave as a catalyst for oxygen/hydrogen peroxide activation and lipid peroxidation. Metalloporphyrins with metals other than iron, such as zinc (Zn protoporphyrin) or tin (Sn protoporphyrin), do not have such a catalytic activity. They can be considered canonical inhibitors of Bach1, which bind to the CP motifs of Bach1. Unfortunately, this class of small molecules is known to inhibit HO-1 activity, minimizing their protective effect to severe adverse effects [256]. The search for HMOX1 activators that do not exhibit HMOX1 enzyme inhibition has been performed by vTv Therapeutics LLC and identified a series of substituted benzimidazole/benzothiazole hits providing almost a 100-fold increase in HMOX1 protein expression [257]. The structural formula of novel HMOX1 activators has been widened to benzoxazoles [258].

Out of the hit compounds identified by vTv Therapeutics LLC. a novel non-electrophilic small molecule, N-(2-(2-hydroxyethoxy)ethyl)-1-methyl-2-((6-(trifluoromethyl)benzo[d]thiazol-2-yl)amino)-1H-benzo[d]imidazole-5-carboxamide (HPPE), has been further validated by MARE-luciferase and Neh2-luciferase assays [207]. HPPE is even more potent than hemin (acting at a lower concentration) at inhibiting Bach1-mediated transcriptional repression of target genes. Further structural analysis coupled with bioinformatic tools points to HPPE’s direct interaction with the heme binding site of Bach1. However, the inhibition of Bach1-mediated gene repression and the induction of Nrf2-regulated ARE genes remains incomplete unless Bach1 is exported from the nucleus to the cytosol. Perhaps this is one of the reasons why, despite significant Nrf2 accumulation in the nucleus of the DA neurons of a PD brain, it is insufficient to activate compensatory neuroprotective genes due to the presence of Bach1-mediated repression [162].

Interestingly, this study demonstrated that the relative occupancy of Nrf2 over Bach1 on the MARE enhancer regions in the promoter region of the HO-1 gene increased by almost eight-fold after HPPE treatment. This value is even higher than that found with hemin or DMF, an alkylating activator of Nrf2. Unlike DMF, HPPE does not alkylate the critical cysteine residues in the Keap1 protein, which binds Nrf2 in the cytosol and prevents nuclear shuttling under basal conditions. As described earlier, DMF, being an electrophile, chemically modifies the thiol groups of Keap1, which releases Nrf2 for translocation into the nucleus. However, this alkylation of DMF is nonspecific and leads to a depletion of GSH in cells. Therefore, the advantage of HPPE is that, due to its non-electrophilic nature, HPPE does not alkylate the thiol groups of Keap1. HPPE does not act as a displacement activator of the Keap1-Nrf2 complex as observed in a fluorescence polarization assay; the investigators confirmed that HPPE, even at high concentrations, does not interfere with the Keap1-Nrf2 interaction through interactions with the Kelch domain of Keap1.

Finally, the study also has characterized the pharmacokinetics of HPPE in vivo: A single oral dose of HPPE (100 mg/kg) in mice has led to an accumulation of this molecule in multiple organs, including the brain, and it was found to persist up to 24 h post-treatment. The abundance of HPPE in tissues is concomitant with the up-regulation of several genes involved in heme degradation and redox regulation, such as HO-1, Gsr, Mafg, Prdx2, Txnrd1, Slc7a11, etc. Finally, in both the pre-treatment and post-treatment paradigms, HPPE administration in MPTP-intoxicated mice exhibits suppression of OS and glial inflammation resulting in the protection of nigral dopaminergic neurons and striatal catecholamines, which is commensurate to the data obtained from the Bach1 knockout mice. These findings demand future investigations of Bach1 inhibition as a therapeutic strategy in chronic genetic models of PD. In addition, the Bach1-regulated non-ARE protective genes, which are less explored in the disease context, require further research to relate their function to PD susceptibility.

## 10. Conclusions

There is an urgent unmet need for safe, potent, and blood–brain barrier-permeable compounds that can orchestrate the activation of the antioxidant and anti-inflammatory signaling cascade within the CNS. The activation of the Nrf2 signaling pathway via Nrf2 activators reduces neurodegeneration in preclinical PD models and, thus, represents a validated target for developing disease-modifying therapeutic interventions for PD. However, a significant limitation of these Nrf2 activators is their highly electrophilic nature, which regrettably renders them unusable in PD treatment. An alternate approach is to develop non-electrophilic Nrf2 activators that rely on destabilizing the Keap1-Nrf2 binding non-covalently. Unfortunately, these activators have low potency and exhibit poor blood–brain barrier permeability. Therefore, currently, all types of Nrf2 activators working via the Nrf2 stabilization mechanism are not ideal candidates for PD treatment. Moreover, Nrf2 stabilization and the subsequent activation induce the expression of Bach1, a transcriptional repressor of Nrf2, via a feedback mechanism. Therefore, without breaking this feedback loop, Nrf2 stabilization through the interruption of the Nrf2-Keap1 interaction is insufficient to combat neurodegeneration in PD. Bach1, the repressor of Nrf2, represents a novel target that intrinsically modulates the pathways common to the Nrf2 axis. Furthermore, the availability of non-electrophilic Bach1 inhibitors represents an excellent opportunity to investigate the therapeutic potential of targeting Bach1 in different preclinical PD models. It is obvious that a combination of the Nrf2 stabilizer with the Bach1 inhibitor could be mutually beneficial [259]. Such a strategy will increase the background level of the Nrf2 protein and simultaneously relieve the inhibition from Bach1, which has the potential to lower the doses of both drugs and allow their continuous administration in the treatment of chronic diseases. Our promising results with genetic and pharmacological approaches to inhibit Bach1 in PD mouse models warrants further investigation into the validation of Bach1 inhibition as an alternate strategy for developing safer and more effective therapeutic interventions for PD and other chronic neurodegenerative diseases.

## Figures and Tables

**Table 1:** Top 25 Bach1-associated DNA binding motifs identified from ChIP-seq data for Bach1 (GSM2086721).

Scheme	Motif Name	LOG *p*-Value	% of Target	Sequences with Motif
1	Bach1(bZIP)	1.00 × 10^−1649^	32.98%	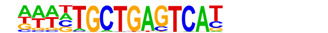
2	NF-E2(bZIP)	1.00 × 10^−1528^	31.44%	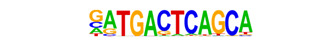
3	Nrf2(bZIP)	1.00 × 10^−1526^	30.48%	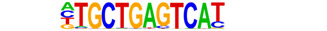
4	Bach2(bZIP)	1.00 × 10^−1345^	38.90%	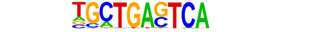
5	Jun-AP1(bZIP)	1.00 × 10^−991^	33.53%	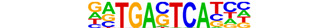
6	MafK(bZIP)	1.00 × 10^−923^	32.31%	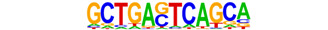
7	Fosl2(bZIP)	1.00 × 10^−906^	35.03%	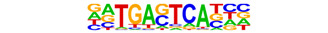
8	Fra2(bZIP)	1.00 × 10^−878^	38.36%	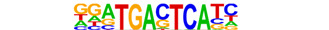
9	JunB(bZIP)	1.00 × 10^−848^	39.74%	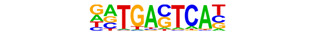
10	AP-1(bZIP)	1.00 × 10^−847^	43.93%	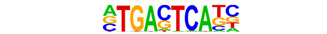
11	Fra1(bZIP)	1.00 × 10^−838^	39.48%	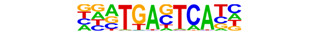
12	BATF(bZIP)	1.00 × 10^−809^	41.34%	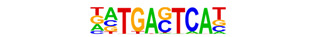
13	Atf3(bZIP)	1.00 × 10^−796^	40.92%	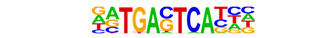
14	MafB(bZIP)	1.00 × 10^−565^	28.69%	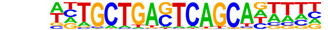
15	MafA(bZIP)	1.00 × 10^−522^	38.58%	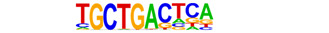
16	MafF(bZIP)	1.00 × 10^−193^	12.78%	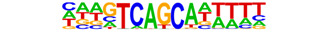
17	Fli1(ETS)	1.00 × 10^−57^	21.71%	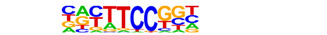
18	ETS1(ETS)	1.00 × 10^−47^	18.44%	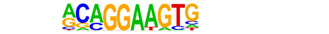
19	ETV1(ETS)	1.00 × 10^−47^	22.96%	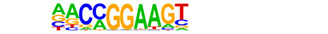
20	PU.1(ETS)	1.00 × 10^−46^	9.67%	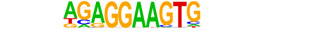
21	ERG(ETS)	1.00 × 10^−45^	24.14%	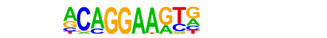
22	Etv2(ETS)	1.00 × 10^−42^	16.07%	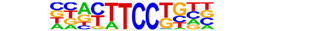
23	GABPA(ETS)	1.00 × 10^−42^	16.81%	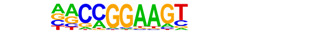
24	SpiB(ETS)	1.00 × 10^−38^	5.76%	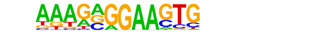
25	ELF1(ETS)	1.00 × 10^−35^	12.23%	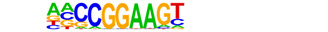

## Supplementary Materials

The following supporting information can be downloaded at: https://www.mdpi.com/article/10.3390/antiox11091780/s1, Figure S1: The absence of Neh2-luciferase reporter activation in the presence of uric acid and inosine in comparison to the well-known Nrf2 activator nordihydroguaiaretic acid (NDGA).
