# Supplementary material for: Harnessing the Therapeutic Potential of the Nrf2/Bach1 Signaling Pathway in Parkinson’s Disease
Source: Antioxidants (Basel). 2022 Sep 9;11(9):1780. doi: 10.3390/antiox11091780 (PMC9495572; doi:10.3390/antiox11091780)
Supplement: Supplementary file 1 [file antioxidants-11-01780-s001.zip › antioxidants-1852981-supplementary.pdf]

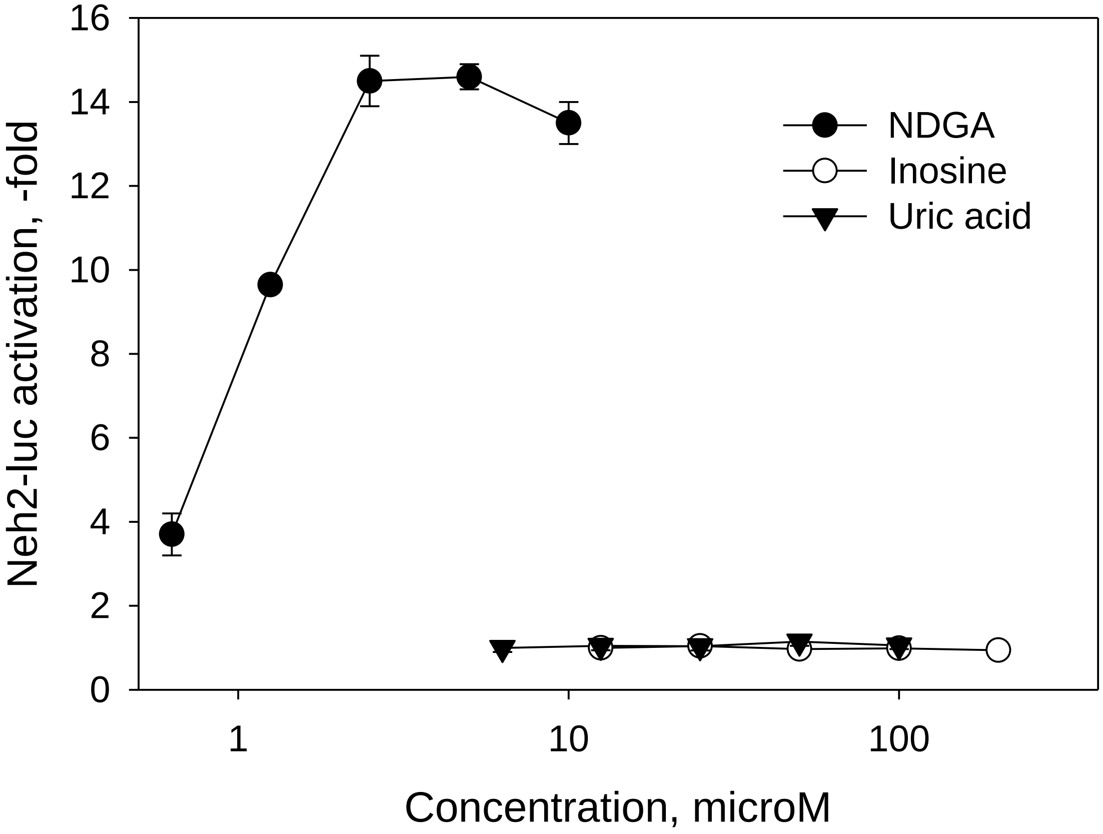

Figure S1: The absence of Neh2-luciferase reporter activation in the presence of uric acid and inosine in comparison to the well-known Nrf2 activator nordihydroguaiaretic acid (NDGA). The assay was performed as we described previously in [163]. Inosine and uric acid stock solutions were prepared in PBS right before the addition to the reporter cells, and the cells were incubated with the compounds for 3 hours before lysis and assay.
